# MOBILITY ABILITIES MEDIATE THE ASSOCIATION BETWEEN A MORE ACTIVE LIFESTYLE WITH MOBILITY DISABILITY

**DOI:** 10.1093/geroni/igae098.0231

**Published:** 2024-12-31

**Authors:** Brittney Lange-Maia, Tianhao Wang, Shahram Oveisgharan, Jeffrey Hausdorff, David Bennett, Aron Buchman

**Affiliations:** Rush University Medical Center, Chicago, Illinois, United States; Rush University Medical Center, Chicago, Illinois, United States; Rush University Medical Center, Chicago, Illinois, United States; Tel Aviv Sourasky Medical Center, Tel Aviv, Tel Aviv, Israel; Rush University Medical Center, Chicago, Illinois, United States; Rush University Medical Center, Chicago, Illinois, United States

## Abstract

Few studies have analyzed sensor-derived metrics of both mobility abilities and total daily physical activity (TDPA). We sought to determine if sensor-derived mobility metrics and quantitative indices of TDPA were independently associated with prevalent and incident mobility disability. Quantitative mobility metrics from 724 ambulatory adults (mean age 82 years, 77.4% female) were derived from a belt-worn sensor. A wrist-worn sensor worn quantified TDPA (up to 10 days). Mobility disability was characterized at baseline and longitudinally using the Rosow-Breslau scale. We examined cross-sectional association of the mobility metrics and TDPA with the odds baseline of disability using logistic regression, and incident disability using Cox models. Several mobility metrics (e.g. step time variability, turning angular velocity) and TDPA were significantly related to prevalent and incident mobility disability when examined in separate models. When examined together, however, the association of TDPA with mobility disability was attenuated. Mediation analysis indicated that the mobility metrics individually mediated between 7%-47% (p< 0.001) of the association between TDPA and cross-sectional mobility disability, and between 20%-40% of the association between TDPA and incident mobility disability. As a group, mobility metrics mediated 58% of the association between TDPA and cross-sectional mobility disability, and 53% of the association with TDPA and incident mobility disability (p< 0.001 for all). Sensor-derived mobility metrics may distinguish different aspects of mobility disability and partially link a more active lifestyle with reduced disability. These findings can inform the design of intervention studies to develop targeted therapies to maintain ambulation and independent living in late life.

